# Non-HDL Cholesterol in Dyslipidemia Screening Among Korean Adolescents: A National Population-Based Survey †

**DOI:** 10.3390/nu18010037

**Published:** 2025-12-22

**Authors:** Hyo-Kyoung Nam, Eungu Kang, Young-Jun Rhie

**Affiliations:** Department of Pediatrics, College of Medicine, Korea University, Seoul 02841, Republic of Korea; muguet@korea.ac.kr (H.-K.N.);

**Keywords:** non-high-density lipoprotein cholesterol, dyslipidemia, adolescent, public health, Korean National Health and Nutrition Examination Survey

## Abstract

Background/Objectives: In pediatric lipid screening, non-high-density lipoprotein (non-HDL) cholesterol has gained relevance as a clinically feasible indicator that is increasingly applied. We assessed the population-level diagnostic performance and the influence of familial lipid risk in Korean adolescents. Methods: A nationally representative sample of 6989 adolescents aged 10–19 years (3684 boys and 3305 girls) was examined from the Korean National Health and Nutrition Examination Survey. We analyzed this nationally representative data to evaluate weighted and adjusted associations among non-HDL cholesterol level at or above 145 mg/dL, dyslipidemia, and parental lipid status. Results: The overall prevalence of dyslipidemia was 28.2% and 26.3% in Korean adolescent boys and girls, respectively. The prevalence of non-HDL cholesterol at or above 145 mg/dL was 7.2% and 9.2% in adolescent boys and girls, respectively. Elevated non-HDL cholesterol levels detected high directly measured or calculated low-density lipoprotein (LDL) cholesterol levels with a sensitivity of 72.9% and 54.0%, and a specificity of 99.7% and 99.8%, respectively, in adolescent boys, and a sensitivity of 77.5% and 64.8%, and a specificity of 99.5% and 99.2%, respectively, in adolescent girls. Compared with total cholesterol, non-HDL cholesterol more accurately identified adolescents with elevated LDL cholesterol in both sexes. These associations remained stable regardless of familial dyslipidemia history in adjusted weighted logistic regression models. Conclusions: Non-HDL cholesterol consistently distinguished adolescents with dyslipidemia independent of familial lipid status. Although a positive parental history increased the risk, non-HDL cholesterol remained a feasible pediatric screening tool in population-based evaluation.

## 1. Introduction

Dietary habits play a central role in the development of dyslipidemia during childhood and adolescence. Increased consumption of energy-dense, highly processed foods, beverages sweetened with added sugars, and diets rich in saturated fat have been associated with adverse lipid profiles, insulin resistance, and early markers of cardiovascular risk. Nutrition-related metabolic alterations in children and adolescents are closely linked to cardiovascular disease later in life, underscoring the importance of timely identification and targeted intervention. Recent national data suggest a growing burden of dyslipidemia among Korean youth. These indicate that the prevalence of elevated non-high-density lipoprotein (non-HDL) cholesterol and other atherogenic lipid abnormalities has increased in parallel with changes in dietary patterns and sedentary behaviors among Korean youth [1,2]. Approximately one in five Korean adolescents shows at least one abnormal lipid parameter, with prevalence exceeding 50% among those with obesity [3,4,5]. From a nutritional epidemiology perspective, lipid-based screening tools such as non-HDL cholesterol offer a practical means of identifying adolescents who may benefit from early dietary and lifestyle interventions. Over the past decades, pediatric dyslipidemia guidelines have been revised from targeted screening to universal screening to reduce the likelihood of missed diagnoses in the pediatric population. The 2011 U.S. National Institutes of Health Heart, Lung, and Blood Institute (NHLBI) Expert Panel, the American Academy of Pediatrics, and the National Lipid Association recommend universal screening for pediatric dyslipidemia [6,7,8]. Following similar principles, the Korean Pediatric Endocrinology Society recommended universal screening for non-HDL cholesterol in pediatric populations aged 9–11 years and 17–21 years [9,10]. However, despite accumulating evidence, a universally accepted cut-off for non-HDL cholesterol in the pediatric population has remained undefined. Analyses evaluating how non-HDL cholesterol performed relative to conventional lipid markers in Korean adolescents are also limited.

Therefore, we aimed to examine the diagnostic utility of non-HDL cholesterol levels in Korean adolescents using nationally representative data. We also investigated the association between parental history of dyslipidemia and adolescent lipid abnormalities, as well as the limitations of relying on family history for screening.

## 2. Participants and Methods

### 2.1. Study Population

This study used data derived from the 2008–2016 Korea National Health and Nutrition Examination Survey (KNHANES), a nationwide program administered by the Korea Centers for Disease Control and Prevention. This nationally representative, cross-sectional cohort utilized a multistage clustered probability sampling method targeting the non-institutionalized South Korean population [11]. The health examination survey was conducted to collect anthropometric and biochemical data. The KNHANES included data of 6989 adolescents (3684 boys and 3305 girls) aged 10–19 years. We excluded participants without lipid data, such as total and HDL cholesterol levels. Blood samples were obtained from the study population following an overnight fast of at least 8 h period. Fasting serum lipid profiles, including total cholesterol, triglyceride, low-density lipoprotein (LDL) cholesterol, and HDL cholesterol levels, were measured.

### 2.2. Definition of Dyslipidemia

Dyslipidemia was defined as meeting at least one pediatric lipid threshold, including total cholesterol at or above 200 mg/dL, triglycerides at or above 130 mg/dL in adolescents aged 10–19 years, LDL cholesterol at or above 130 mg/dL, or HDL cholesterol below 40 mg/dL. A non-HDL cholesterol at or above 145 mg/dL was used as the primary cutoff value. These criteria were based on the guidelines of the NHLBI Expert Panel recommendations [6] and Korean clinical practice guidelines for dyslipidemia in children and adolescents [12]. These cutoff values are identical across the guidelines, supporting their applicability to Korean adolescents and facilitating comparison with international studies. Serum non-HDL cholesterol was calculated as total cholesterol minus HDL cholesterol, whereas LDL cholesterol was calculated using the Friedewald equation (LDL cholesterol = Total cholesterol − HDL cholesterol − Triglyceride/5). Calculated LDL cholesterol was used for the entire cohort, as directly measured LDL cholesterol was available only in a subset of participants. To assess the consistency of our findings, we conducted a further analysis of a limited participants with directly measured LDL cholesterol.

### 2.3. Statistical Analysis

Non-HDL cholesterol at or above 145 mg/dL was evaluated for its ability to identify abnormal lipid status using sensitivity and specificity estimates. Paired binary classifications were compared using McNemar’s test, using established cut-off thresholds for total cholesterol, triglycerides, and calculated/directly measured LDL cholesterol as reference standards. Differences in the discriminative abilities of total cholesterol and non-HDL cholesterol to identify elevated LDL cholesterol levels (≥130 mg/dL) were determined using receiver operating characteristic (ROC) curves. The areas under the ROC curve (AUC), with 95% confidence intervals (CIs), were estimated separately according to sex. We also assessed whether non-HDL cholesterol maintains consistent screening performance independent of family history to consider its potential role as a universal screening. A history of parental dyslipidemia was coded as positive if either parent had dyslipidemia, and all others were classified as negative. Weighted logistic regression analysis was used to estimate the association between parental and adolescent dyslipidemia. A multiple logistic regression model was additionally adjusted for parental dyslipidemia, age, sex, and body mass index. Adjusted odds ratios (ORs) with 95% CIs were standardized to 1.0 in the reference model to enable comparison across models.

Statistical analyses were performed using SPSS (version 20.0; IBM Co., Armonk, NY, USA). The Kolmogorov–Smirnov test was used to examine the distribution of continuous variables. Accordingly, data are reported as means with standard deviation or as medians with interquartile ranges, as appropriate based on the results of the normality test. Categorical variables were presented as numbers (percentage). All prevalence estimates and percentiles incorporated the complex survey design, including stratification, sampling weights, and primary sampling units, to ensure nationally population-representative estimates for Korean adolescents aged 10–19 years with available lipid measurements. A *p*-value < 0.05 was considered statistically significant.

## 3. Results

In total, 6989 adolescents aged 10–19 years were analyzed, including 3684 boys (52.7%) and 3305 girls (47.3%), with a mean age of 14.6 ± 2.8 years. In boys, the median total cholesterol was 153.0 (137.0–172.0) mg/dL, whereas it was 161.0 (146.0–179.0) mg/dL in girls. The median triglyceride level was 71.5 (51.0–102.0) mg/dL in boys and 76.0 (56.0–103.0) mg/dL in girls. Calculated and directly measured LDL cholesterol levels were 86.8 (73.1–102.7) mg/dL and 89.0 (75.0–108.8) mg/dL in boys, and 91.4 (78.4–106.6) mg/dL and 96.0 (83.0–113.0) mg/dL in girls, respectively. The median non-HDL cholesterol was 103.0 (88.0–121.0) mg/dL in boys and 108.0 (94.0–125.0) mg/dL in girls. The HDL cholesterol levels ranged from 44 to 57 mg/dL across all adolescents. Overall, 26.9% of adolescents met the criteria for dyslipidemia, and elevated lipid parameters—including total cholesterol, triglycerides, calculated LDL cholesterol, directly measured LDL cholesterol, and non-HDL cholesterol at or above 145 mg/dL—were observed in 7.3%, 13.2%, 5.3%, 8.6%, and 8.2% of the adolescents, respectively (Table 1).

The weighted prevalence of non-HDL cholesterol at or above 145 mg/dL was 7.2% (95% CI, 6.2–8.2) among boys and 9.2% (95% CI, 8.1–10.4) among girls. Using this cut-off, non-HDL cholesterol demonstrated consistently high accuracy in identifying adolescents with elevated lipid parameters. Sensitivity and specificity for detecting elevated directly measured or calculated LDL cholesterol and total cholesterol were 68.5% and 98.1%, 75.0% and 99.6%, and 59.6% and 99.5%, respectively (all *p* < 0.001). When stratified by sex, the identification of elevated total cholesterol yielded sensitivity and specificity of 63.0% and 98.9% in boys, and 73.9% and 97.2% in girls (both *p* < 0.001). For calculated or directly measured LDL cholesterol at or above 130 mg/dL, non-HDL cholesterol ≥ 145 mg/dL identified nearly all affected adolescents, with specificity of 99.7 and 99.8% in boys and 99.2 and 99.5% in girls, respectively (Table 2).

In the assessment of elevated LDL cholesterol, total cholesterol showed modest sensitivity with consistently high specificity in both sexes. Sensitivity and specificity were 55.6% and 99.0% in boys, and 60.6% and 99.2% in girls, respectively. When compared with total cholesterol, non-HDL cholesterol provided marginally greater specificity across sexes; however, the difference was statistically significant only in boys (boys: 99.0% vs. 99.8%, *p* = 0.020; girls: 99.2% vs. 99.2%, *p* = 0.140) (Table 3).

**Figure 1:**
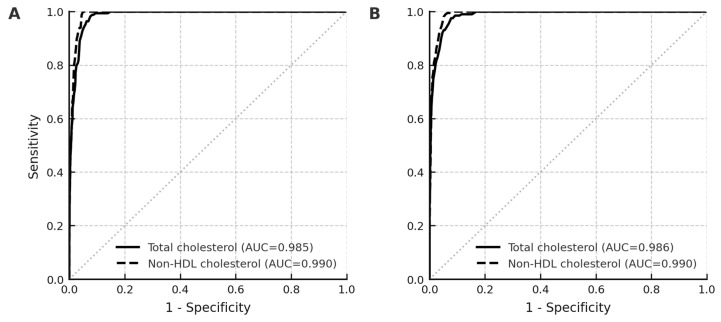
Discriminative performance of total cholesterol and non-HDL cholesterol for elevated calculated LDL cholesterol (≥130 mg/dL) using receiver operating characteristic curves. Total cholesterol and non-HDL cholesterol are illustrated using solid black and dashed gray lines, respectively. Sex-stratified analyses are presented for boys (**A**) and girls (**B**).

ROC analyses showed comparable discriminatory ability of total cholesterol and non-HDL cholesterol for elevated calculated LDL cholesterol (≥130 mg/dL) in both boys and girls. Among boys, the AUC was 0.985 (95% CI, 0.981–0.989) for total cholesterol and 0.990 (95% CI, 0.987–0.992) for non-HDL cholesterol, with a small but statistically significant difference *(p* = 0.020). In girls, corresponding AUCs were 0.986 (95% CI, 0.982–0.990) and 0.990 (95% CI, 0.987–0.993), respectively, with no significant difference between the two (*p* = 0.140) (Figure 1). Across analyses using calculated or directly measured LDL cholesterol as reference, non-HDL cholesterol achieved marginally higher AUC values than total cholesterol.

Adolescents having at least one parent with a history of dyslipidemia had a higher prevalence of adolescent dyslipidemia than those without parental dyslipidemia. When parental dyslipidemia was defined using calculated LDL cholesterol, the prevalence of adolescent dyslipidemia was 28.7% (26.7–30.7%) in the positive family history group and 26.1% (24.6–27.7%) in the negative family history group, corresponding to an OR of 1.14 (95% CI: 1.138–1.141, *p* < 0.001). Using measured LDL cholesterol to define parental dyslipidemia, the prevalence of adolescent dyslipidemia was 31.0% (27.5–34.5%) vs. 26.5% (25.2–27.8%), with an OR of 1.25 (95% CI: 1.246–1.251, *p* < 0.001). In the weighted multiple logistic regression model that included both non-HDL cholesterol ≥ 145 mg/dL and parental dyslipidemia, OR was 1.002 (95% CI: 1.000–1.005, *p* < 0.001).

## 4. Discussion

This study showed that non-HDL cholesterol at or above 145 mg/dL provided a valuable diagnostic performance for detecting elevated LDL cholesterol and other dyslipidemia components in a nationally representative cohort of Korean adolescents. Compared with total cholesterol, non-HDL cholesterol showed superior or at least non-inferior discriminative ability for calculated and directly measured LDL cholesterol across both sexes. Furthermore, even after accounting for parental dyslipidemia in weighted regression models, non-HDL cholesterol at or above 145 mg/dL remained the dominant predictor of dyslipidemia.

Dyslipidemia during childhood poses an increasing public health challenge independent of obesity, owing to its strong links with adverse cardiovascular outcomes later in life. Unhealthy dietary patterns, such as diets high in saturated fats, trans fats, and added sugars in childhood, have been shown to mediate lipid abnormalities and contribute to early vascular changes, insulin resistance, and rising burden of cardiovascular risk in adulthood. Taiwanese adolescents consuming large quantities of sugar-containing beverages had greater odds of metabolic syndrome. Children adhering to a Westernized diet had a higher prevalence of abnormal lipids even after controlling physical activity [13]. These findings underscore the importance of preventive interventions through dietary counseling. Targeted screening in high-risk pediatric populations missed a substantial number of patients with clinically significant dyslipidemia [14]. LDL cholesterol reflects only a portion of atherogenic lipoproteins and typically requires fasting conditions to ensure accurate measurement. In contrast, non-HDL cholesterol captures the full spectrum of atherogenic apolipoprotein B-containing particles, and childhood levels of this marker demonstrated greater predictive value than LDL cholesterol for adult dyslipidemia [15,16,17], carotid intima-media thickness [18], and other cardiovascular risk factors unrelated to lipid profiles [19]. Puberty is known to influence lipid metabolism, with dynamic changes in lipid fractions occurring across pubertal stages. Longitudinal studies show that non-HDL cholesterol levels in childhood and adolescence often track into adulthood, which supports the stability of non-HDL cholesterol levels across developmental stages [16,20,21].

Dyslipidemia cut-off values were applied uniformly across ages 10–19 years in accordance with current pediatric guidelines, which do not recommend age- or puberty-specific cutoffs for non-HDL cholesterol. In our study, the prevalence of elevated total cholesterol, triglycerides, calculated LDL cholesterol, directly measured LDL cholesterol, and non-HDL cholesterol at or above 145 mg/dL were observed in 5.7%, 13.8%, 4.2%, 8.2%, and 7.2% in boys, and in 9.1%, 12.5%, 9.1%, 6.4%, and 9.2% in girls, respectively. Despite the differences in the study period, the lipid parameters and prevalence of dyslipidemia observed in our study were broadly in line with previous reports based on the same KNHANES population [22,23]. And identical cut-off values are recommended by both international and Korean pediatric guidelines, our prevalence estimates are comparable with those reported in other population-based studies.

Children with high non-HDL cholesterol but normal LDL cholesterol presented significantly higher atherosclerotic cardiovascular outcomes in adulthood [24]. In contrast, those who had elevated non-HDL cholesterol during childhood but achieved normal levels by adulthood exhibited reduced cardiovascular risk, underscoring the importance of timely detection and management. Despite the recognized importance of universal lipid screening, current rates remained suboptimal [25]. Accordingly, we examined non-HDL cholesterol, which is not affected by fasting, and found that its diagnostic performance was comparable to that of total cholesterol. Although the differences in AUC values between total cholesterol and non-HDL cholesterol were numerically small in both sexes, only boys showed a statistically significant advantage for non-HDL cholesterol. These differences indicate that non-HDL cholesterol had a slightly but consistently better discriminative ability for dyslipidemia screening than that of LDL cholesterol. The application of population weights ensured that the findings could be generalized to the Korean adolescent population. The similarity between the weighted and unweighted estimates suggests that the diagnostic reliability of non-HDL cholesterol is stable across analytical methods.

Several previous epidemiological studies have shown that relying solely on a positive family history is insufficient for identifying dyslipidemia in children and adolescents [26,27]. In another Korean study, the sensitivity for identifying dyslipidemia increased from 4.3% to 17.6% when three generations of family history were considered rather than parents only [23]. Although these data reaffirm that a positive family history is associated with an increased risk of dyslipidemia in children and adolescents, poor sensitivity results highlight that reliance on family history alone would result in the failure to identify a substantial proportion of affected youths. Consistent with the poor sensitivity of family history-based screening findings, our study also observed a higher prevalence of lipid abnormalities among participants with a positive family history than among those without a positive family history. The prevalence of abnormal lipid markers remained significant even among those without a reported family history, suggesting that a family history-driven approach would fail to identify many at-risk individuals. A previous study reported that approximately 30.1% of children without any known risk factors had abnormal lipid profiles, and targeted screening alone is estimated to overlook 30–60% of pediatric dyslipidemia cases [25]. This limitation may be even more pronounced in Korean adolescents, where parental awareness and diagnosis rates of dyslipidemia remain relatively low, and routine lipid screening in adults is often delayed until midlife [28]. As a result, Korean adolescents may be less likely to report an accurate family history, leading to further underestimation when family history-based screening strategies are applied.

Familial dyslipidemia represents a stable, genetically driven determinant of lipid levels that is relatively less affected by short-term lifestyle changes, and non-HDL cholesterol has been shown to effectively detect heritable atherogenic lipid patterns [29]. The diagnostic accuracy of non-HDL cholesterol was nearly identical between adolescents, irrespective of a parental dyslipidemia history. This was evident in multivariable models and adjusted prevalence estimates. Multiple analyses stratified by parental dyslipidemia indicated that the diagnostic performance of non-HDL cholesterol in identifying dyslipidemia remained unchanged. Non-HDL cholesterol identifies the majority of lipid-related risks independent of family history, whereas parental lipid status may help further stratify long-term risk in borderline cases.

Our findings should be interpreted considering several limitations. First, because of the cross-sectional study, we could not assess longitudinal outcomes or direct cardiovascular events. Second, LDL cholesterol levels for all participants were calculated and not directly measured; however, we confirmed consistent results using directly measured LDL cholesterol levels where available. Third, confounding factors such as physical activity, lifestyle, dietary factors or socioeconomic status could not be fully excluded. Nevertheless, the large sample size, standardized measurements, and use of nationally representative weights strengthened the validity of our conclusions.

## 5. Conclusions

In conclusion, non-HDL cholesterol may be considered an informative screening tool for identifying adolescents at increased risk of dyslipidemia in diverse clinical settings. It is also useful in adolescents regardless of familial lipid risk. Thus, given its simplicity and clinical feasibility, non-HDL cholesterol may serve as a useful screening marker in line with recent guidelines. Nonetheless, longitudinal studies incorporating pubertal state and using directly measured LDL cholesterol are needed to confirm its utility over time.

## Figures and Tables

**Table 1:** Clinical and biochemical characteristics of participants and the prevalence of dyslipidemia.

	Boys	Girls	Total
Unweighted number (%)	3684 (52.7%)	3305 (47.3%)	6989 (100%)
Age, years	14.6 ± 2.8	14.6 ± 2.8	14.6 ± 2.8
Dyslipidemia (%)	28.2 (26.4–29.9)	26.3 (24.6–28.0)	26.9 (25.7–28.2)
Total cholesterol ≥ 200 mg/dL (%)	5.7 (4.8–6.6)	9.1 (8.0–10.3)	7.3 (6.6–8.0)
Non-HDL cholesterol ≥ 145 mg/dL (%)	7.2 (6.2–8.2)	9.2 (8.1–10.4)	8.2 (7.4–8.9)
Measured LDL cholesterol ≥ 130 mg/dL (%)	8.2 (5.1–11.3)	9.1 (5.9–12.4)	8.6 (6.4–10.9)
Calculated LDL cholesterol ≥ 130 mg/dL (%)	4.2 (3.5–5.0)	6.4 (5.4–7.4)	5.3 (4.6–5.9)
Triglycerides ≥ 130 mg/dL (%)	13.8 (12.5–15.1)	12.5 (11.2–13.8)	13.2 (12.3–14.1)
HDL cholesterol < 40 mg/dL (%)	14.4 (13.0–15.7)	8.4 (7.3–9.5)	11.6 (10.7–12.4)

Data are expressed as mean ± standard deviation for normally distributed variables and as median (interquartile range) for non-normally distributed variables. HDL, high-density lipoprotein; LDL, low-density lipoprotein.

**Table 2:** Sensitivity and specificity of non-HDL cholesterol for dyslipidemia according to sex.

Criteria	Cholesterol Cut-Off Values	Number (%)	Sensitivity	Specificity
Boys	Total cholesterol ≥ 200 mg/dL	183 (5.0%)	63.0 (55.7–70.2)	98.9 (98.4–99.4)
	Triglycerides ≥ 130 mg/dL	137 (3.7%)	56.1 (47.9–64.4)	88.8 (87.4–90.2)
	Measured LDL cholesterol ≥ 130 mg/dL	33 (0.9%)	72.9 (62.7–83.1)	99.7 (99.1–100.0)
	Calculated LDL cholesterol ≥ 130 mg/dL	152 (4.1%)	54.0 (45.6–62.3)	99.8 (99.6–99.9)
Girls	Total cholesterol ≥ 200 mg/dL	207 (6.3%)	73.9 (66.7–83.1)	97.2 (96.3–98.0)
	Triglycerides ≥ 130 mg/dL	105 (3.2%)	36.5 (28.0–44.9)	89.9 (88.5–91.4)
	Measured LDL cholesterol ≥ 130 mg/dL	36 (1.1%)	77.5 (62.5–92.4)	99.5 (98.9–100.0)
	Calculated LDL cholesterol ≥ 130 mg/dL	182 (5.5%)	64.8 (56.5–73.2)	99.2 (98.7–99.7)

Data are expressed as number (%) or mean ± 95% confidence interval (CI). HDL, high-density lipoprotein; LDL, low-density lipoprotein.

**Table 3:** Sensitivity and specificity of non-HDL cholesterol and total cholesterol for elevated LDL cholesterol according to sex.

Sex	Cholesterol Cut-Off Values	Sensitivity (%)	Specificity (%)
Boys	Total cholesterol ≥ 200 mg/dL	55.6 (45.7–65.4)	99.0 (98.6–99.4)
	Non-HDL cholesterol ≥ 145 mg/dL	54.0 (45.6–62.3)	99.8 (99.6–99.9)
Girls	Total cholesterol ≥ 200 mg/dL	60.6 (51.8–69.5)	99.2 (98.8–99.6)
	Non-HDL cholesterol ≥ 145 mg/dL	64.8 (56.5–73.2)	99.2 (98.7–99.7)

Data are expressed as number (%) or mean ± 95% confidence interval (CI). HDL, high-density lipoprotein; LDL, low-density lipoprotein.

## Data Availability

The data presented in this study are openly available in KNHANES repository at https://knhanes.kdca.go.kr/knhanes/eng/main.do (accessed on 1 November 2025).

## Abbreviations

The following abbreviations are used in this manuscript:

Non-HDL cholesterol	Non-high-density lipoprotein cholesterol
NHLBI	National Institutes of Health Heart, Lung, and Blood Institute
LDL cholesterol	Low-density lipoprotein cholesterol
ROC	Receiver operating characteristic
CIs	Confidence intervals
ORs	Odds ratios
